# Editorial: Contribution of Uremic Compounds to Cardiorenal Syndrome Development

**DOI:** 10.3389/fphys.2022.877182

**Published:** 2022-05-18

**Authors:** Marcela Sorelli Carneiro-Ramos, Gema Ruiz-Hurtado, Adalberto Vieyra

**Affiliations:** ^1^ Laboratory of Cardiovascular Immunology, Center of Natural and Human Sciences (CCNH), Federal University of ABC, Santo André, Brazil; ^2^ Cardiorenal Translational Laboratory, Institute of Research i+12, Hospital Universitario 12 de Octubre, Madrid, Spain; ^3^ CIBER-CV, Hospital Universitario 12 de Octubre, Madrid, Spain; ^4^ Carlos Chagas Filho Institute of Biophysics and National Center of Structural Biology and Bioimaging/CENABIO, Federal University of Rio de Janeiro, Rio de Janeiro, Brazil; ^5^ Graduate Program in Translational Biomedicine/BIOTRANS, University of Grande Rio/UNIGRANRIO, Duque de Caxias, Brazil

**Keywords:** uremic compounds, cardiorenal syndrome (CRS), renal diseases, cardiovascular disease (7), chronic kidney disease

Within an extensive roster of uremic toxins, the protein-bound uremic toxins, (PBUT) *p*-cresyl sulfate (PCS) and indoxyl sulfate (IS), deserve special mention. These compounds originate from the intestinal microbiota during metabolism of tyrosine/phenylalanine and tryptophan, respectively. IS causes immune dysfunction with activation of pro-inflammatory macrophages and the release of IL-6 and TNF-α, whereas PCS appears to be involved in activating the renin-angiotensin-aldosterone system (RAAS), epithelial-mesenchymal transition, fibrosis, and nephrosclerosis. The data reviewed by Falconi *et al.* demonstrate that renal pathologies can also aggravate renal lesions, in addition to causing damage to the heart and various other organs. Novel insights offered by the Falconi *et al.* review include the observation that the cardiorenal syndrome resulting from accumulation of uremic toxins is not restricted to the kidney and heart; it impacts almost all organs and tissues. Finally, the analysis of intracellular mechanisms of uremic toxins reveals changes in genes, microRNAs, cell cycle, oxidative stress, and cell-to-cell communication. Figure 1, taken from the Falconi et al. review, summarizes this panorama.

**FIGURE 1:**
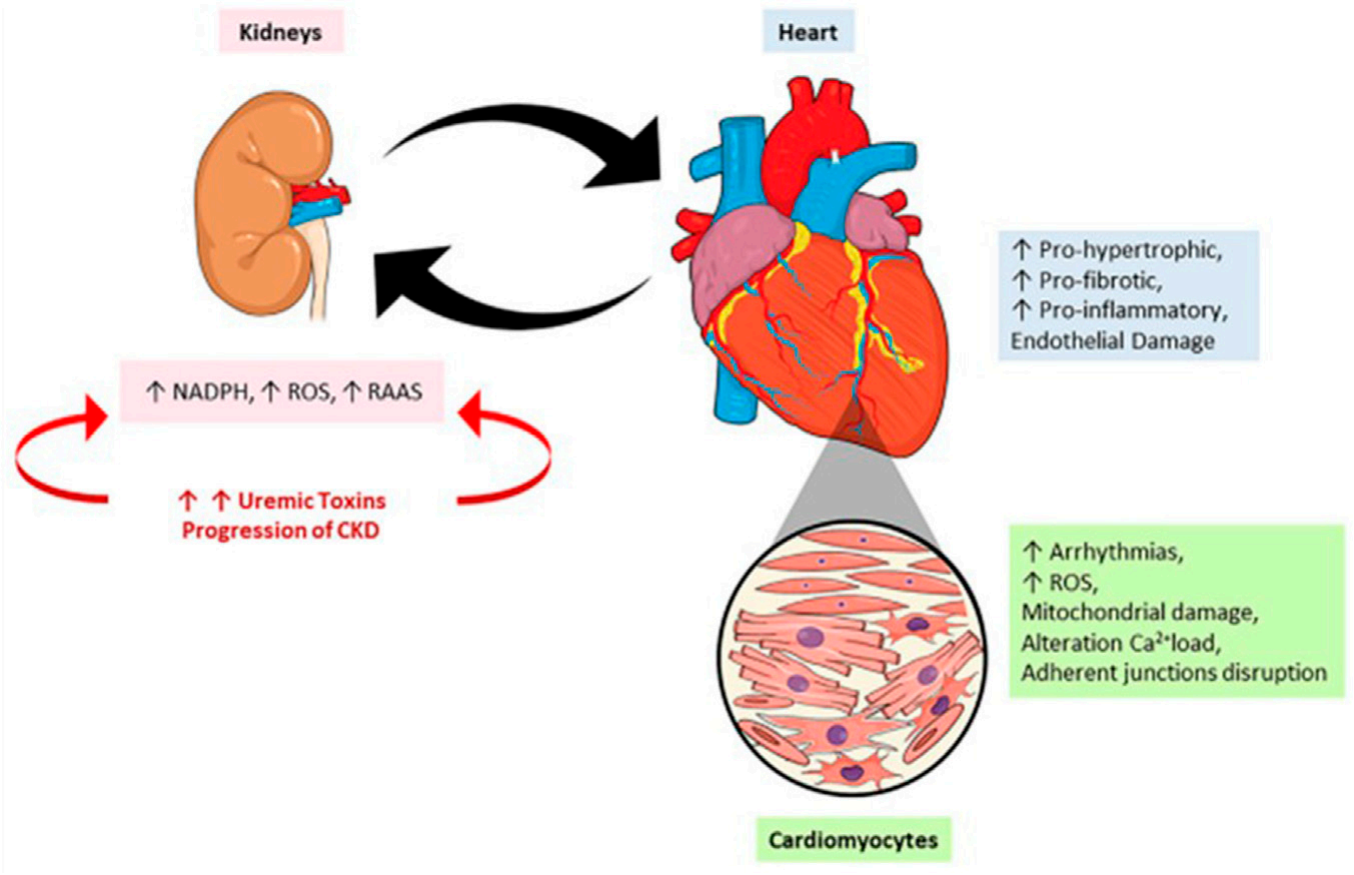
The crosstalk between heart and kidney: contributions to uremic toxins. During the progression of a renal lesion there is an increase in the circulatory levels of uremic toxins accompanied by an increase in renin-angiotensin-aldosterone system (RAAS) activity and reactive oxygen species (ROS). On the other hand, the heart responds to kidney injury by the onset of pro-oxidant, pro-inflammatory effects leading to mitochondrial damage, alterations in calcium loading, arrhythmias, and cardiac remodeling.

The original research articles by Huang et al. and Hamza et al. explore the role of increased renal venous pressure (RVP), which is an important but not fully understood pathophysiological component of simultaneous heart and kidney failure. Huang et al. first investigated the role of RAAS and the autoregulation of renal blood flow (RBF) in the renal hemodynamic response to increased RVP. These parameters are important in connection with the data reviewed by Falconi *et al.* because elevated RVP reduces glomerular filtration rate (GFR) and RBF; consequently, this reduces the clearance of uremic toxins in CKD patients with cardiovascular lesions. Key information regarding the role of RAAS is that, in their study, Huang *et al.* demonstrated that the RVP-induced reduction in renal vascular conductance (RVC) was prevented in the absence of angiotensin II (Ang II) modulation or Ang II clamp, *i.e.* Ang I infusion after inhibition of angiotensin converting enzyme (ACE) with Enalapril. Since they found the same response in the absence of Ang II (*i.e*. during inhibition of ACE alone), they inferred that RVP-induced vasoconstriction is dependent on the modulation of RAAS and its central peptide Ang II by other compounds, probably nitric oxide (NO). It is noteworthy, as reviewed by Falconi *et al.*, that uremic toxins impair endothelium-dependent vasodilatation, as a result of decreased formation of NO by NO synthase, whose activity and expression are downregulated by uremic serum, IS, and other toxins. This may mean that there are additional links between the actions of accumulated uremic toxins and altered renal venous circulation.

In the cardiovascular alterations that occur secondary to an altered RVP, the arterial baroreceptor reflex gain (Δheart rate/Δmean arterial pressure) was also modified, as they demonstrated in Figure 2 from the Huang *et al.* study, suggesting that other mediators interact with Ang II and its effects. As in the case of the results reviewed by Falconi *et al.*, the data from Huang *et al.* support the view that kidney diseases can affect not only the heart but also the cardiovascular system as a whole. Again, the increased RVP that led to elevation of preglomerular, efferent arteriolar vasoconstriction, and increased tubular pressure would affect depuration of uremic toxins. This is another point of convergence between the ideas developed in both articles.

The more recent contribution by the same group (Hamza et al.) demonstrated that the chronic combined dysfunction of the heart and kidney exacerbates the renal venous pressure-induced suppression of renal function in rats. Using a surgical model of subtotal 5/6 nephrectomy in combination with induced myocardial infarction and consequent cardiac dysfunction and acute elevation of RVP, they demonstrated reduced GFR–a scenario for impaired depuration of toxins–and a fall in RBF. Interestingly, GFR was maintained in sham-operated rats in response to acute RVP elevation, a demonstration that simultaneous renal and cardiac tissular alterations negatively impact the renal circulation. Possibly, as they suggest, a varied spectrum of “cardiorenal connectors” is involved in these combined pathological interactions between the two organs with systemic repercussions that may trigger RAAS activation together with salt retention. This vicious circle would worsen the cardiac and renal dysfunctions.

The work presented by Falconi *et al.* shows a very comprehensive review of uremic toxins, definition, classification, and their effects on the kidney-heart axis in a very comprehensive way. In a complementary way, Navarro-Garcia et al. presented a very interesting view regarding mineral bone disorders (MBD) as a possible link between renal dysfunction and the development of cardiovascular alterations. The mineral disturbance is due, in part, to the increase in serum phosphate levels seen in patients with CKD. On the other hand, the Klotho/FGF-23 axis is an important marker of kidney damage, and FGF-23 is a critical factor in developing cardiac hypertrophy and dysfunction, observed in patients with renal disease. These last observations suggest new promising directions for the treatment of cardiorenal syndromes.

## Abbreviations

NADPH, reduced nicotinamide adenine dinucleotide phosphate; ROS, reactive oxygen species; RAAS, renin-angiotensin-aldosterone system.

